# Editorial: Gut microbiota as a therapeutic target in neuropsychiatric disorders: current status and future directions

**DOI:** 10.3389/fnins.2023.1198291

**Published:** 2023-05-23

**Authors:** Hualin Cai, Xia Chen, Aurelijus Burokas, Rafael Maldonado

**Affiliations:** ^1^Department of Pharmacy, Second Xiangya Hospital of Central South University, Changsha, China; ^2^The Institute of Clinical Pharmacy, Central South University, Changsha, China; ^3^International Research Center for Precision Medicine, Transformative Technology and Software Services, Hunan, China; ^4^Beijing Tiantan Hospital, Capital Medical University, Beijing, China; ^5^Department of Biological Models, Institute of Biochemistry, Life Sciences Center, Vilnius University, Vilnius, Lithuania; ^6^Laboratory of Neuropharmacology-Neurophar, Department of Medicine and Life Sciences, Universitat Pompeu Fabra (UPF), Barcelona, Spain; ^7^Hospital del Mar Medical Research Institute (IMIM), Barcelona, Spain

**Keywords:** gut microbiota, psychotropic drugs, prebiotics, probiotics, neuropsychiatry disorder

This Research Topic, started with the aim to explore the roles and provide further insights for gut microbiota involved in the pathophysiology and treatment of neuropsychiatric disorders, has now included 12 research and review articles. There is an undoubtedly complex communication between the microorganisms in the gastrointestinal tract and the central nervous system (CNS), since the gut microbiota constitutes the major component of the “gut-brain axis” that includes various pathways that enable communication between the gut and the CNS. Among those pathways, although emerging evidence implicates the dysregulated kynurenine pathway in the pathophysiology of mood disorders, whether the metabolites of the kynurenine pathway can serve as modulators linking the gut microbiota with the CNS is largely unclear. Bibliometric analysis is a statistical method can be applied to quantitatively analyze and visualize scientific output, research hotspots, and developing trends, by utilizing public literature databases (Zhang et al., 2020). With the help of bibliometric analytic method, here Zhu X. et al. comprehensively evaluate the general aspects and future trends connecting kynurenine pathway and the gut microbiota in research of mood disorders. The mounting evidence suggests that the balance between kynurenic acid and quinolinic acid plays a key role in the pathophysiology of mood disorders under the control of the gut microbiota. Researches focusing on the gut microbiota-brain axis were also identified as frontiers and hotspots in this field. Under the circumstances, many recent studies have reported that the gut microbiota influences cognitive function through the gut-brain axis, which is involved in the pathophysiology of neurodegenerative and mental disorders, including Alzheimer's disease (Kesika et al., 2021) and schizophrenia (Zeng et al., 2021). Similarly, in this topic Dai et al. summarized the emerging evidence of alterations in the gut microbial composition of patients with bipolar disorder, suggesting that gut microbial dysbiosis contributes to disease progression and cognitive impairment. They indicated that gut microbiota modulates neurological function in the brain through various pathways such as productions of microbial-derived metabolites, neurotransmitters, and gastrointestinal hormones, affecting patients' cognitive function. Meanwhile, gut microbiota-related end-products may influence Parkinson's disease pathology through creating peripheral and systemic inflammatory environments (Perez-Pardo et al., 2017). Here, Hill et al. collected the associative evidence from case-control studies and functional evidence from animal models which support for microbiota causing Parkinson's disease. They concluded that two main routes linking gut microbiota can influence Parkinson's disease pathophysiology, the neural and humoral routes. The neural route involves alpha-synuclein misfolding peripherally in the enteric nerves which can then be transported to the brain via the vagus nerve. The humoral route involves transportation of bacterial products and proinflammatory cytokines from the gut via the circulation which can cause central alpha-synuclein misfolding by inducing neuroinflammation.

Except for the interactions between gut microbiota and the pathophysiological mechanisms of these neuropsychiatric disorders, several lines of data demonstrate that complex interactions with the gut microbiota may also explain some of the cognitive and/or metabolic side effects of certain psychiatric medications. Herein, Zhu Z. et al. explored the mechanism by which olanzapine-induced lipid disturbances through the gut microbiota-brain axis. They found that olanzapine increased the *Firmicutes/Bacteroides* (F/B) ratio in the gut, which can be even aggravated by subphrenic vagotomy, reduced the abundance of short-chain fatty acids and 5-hydroxytryptamine levels in the rat cecum, and increased the gene and protein expression of the appetite-related neuropeptide Y/agouti-related peptide in the hypothalamus. These results suggest that abnormal lipid metabolism caused by olanzapine may be closely related to the vagus nerve-mediated gut microbiota-brain axis. On the one hand, the data reinforce the idea that antipsychotic drugs induced changes in these gut microbiota-derived molecules can directly activate the vagus nerve or be transported into the brain to influence appetite (Oliphant and Allen-Vercoe, 2019). On the other hand, accumulated evidence implied that these molecules can also regulate related lipid metabolism via peripheral signaling pathways. Keep this in mind, here Chen et al. reviewed the mechanisms of second-generation antipsychotic drug-induced disorders of lipid metabolism mediated by the gut microbiota, not just from the CNS but also from the periphery. They reported that antipsychotic related peripheral regulations of lipid metabolism can be exerted by shorts-chain fatty acids, bile acids, leptin and glucagon-like peptide 1. The major signaling pathways including AMPK, MAPK, PI3K/Akt/mTOR, and cAMP/PKA/CREB-P work systematically in concert to regulate fatty acid oxidation, fatty acid synthesis, protein synthesis, mitochondrial oxidative phosphorylation and energy metabolism. Apart from antipsychotic-induced metabolic side effects, surgery and anesthesia medication related perioperative neurocognitive dysfunction (PND) is receiving increasing attention nowadays, and there is a strong link between gut microbiota and PND that depends on the gut-brain axis. Here, Lu et al. reviewed the potential mechanisms of the dysbiosis of gut microbiota underlying PND. They concluded that surgery and anesthesia may disrupt gut microbiota homeostasis in a direct or indirect manner. Then gut microbiota dysbiosis leads to altered levels of neurotransmitters, causes abnormal gut microbiota-host co-metabolites, and affects intestinal mucosal permeability (“leaky gut”), resulting in an increased entering of Aβ proteins into bloodstream and subsequent neuroinflammation. Related to the above, more preclinical and clinical researches are needed in the future to focus on the role of specific gut microbiome and specific targets of gut microbiota, in order to provide new approaches for coping with these neuropsychiatric disorders and side effects of certain medications.

As a matter of fact, probiotics play an increasingly important role in acting as alternative medicines/drugs, filling the gap in the therapeutics of different psychopathologies, and contributing to the pharmacological response with fewer adverse effects (Morkl et al., 2020). Herein, Rehman et al. investigated the effects of probiotic formulations (*Lactobacillus fermentum* NMCC-14 and *Bacillus clausii*, 10^10^ colony forming unit/day/animal, per oral) in acute (up to day 7) and subacute (days 8–14) restraint-stressed and normal mice through behavioral paradigms. The results showed improvements in behavioral tests, cortisol and adrenocorticotropic hormone levels, hippocampal neurodegenerative status, monoamine levels, and mRNA expression of dopamine receptor subtypes in probiotic-treated restraint-stressed mice. They concluded that in comparison, *Bacillus clausii* showed greater stress suppressant activity than *Lactobacillus fermentum* NMCC-14. However, both probiotics can be better and safer therapeutic alternatives for stress-related ailments than currently available drugs. Accordingly, another study conducted by Zhang et al. has indicated that the chronic stress-induced behavioral disorders can be ameliorated by treatment with probiotic *Clostridium butyricum* (*C. butyricum*). The data showed that chronic foot shock stress resulted in downregulation of tissue-type plasminogen activator (tPA) but upregulation of plasminogen activator inhibitor 1 (PAI-1), which could contribute to the decrease in BDNF by reduced conversion from proBDNF to BDNF in the hippocampus, and *C. butyricum* RH2 attenuated stress-induced behavior via inhibiting the expression of PAI-1 without changing tPA. These findings suggest that targeting PAI-1 could be an innovative strategy for the development of new drugs to counter the effects of stress. From biological perspective, stressful conditions can result in increased levels of oxidative stress markers (Casado et al., 2011), whereas persistent state of oxidative stress may in turn lead to vulnerability to neuropsychiatric disorder produced by stressful life events (Bouvier et al., 2017). Herein, Zeng et al. investigated the effects of probiotic supplementation on plasma oxidative stress-related biomarkers and different domains of clinical symptom in patients suffering from bipolar disorder. The results showed that after 3 months of intervention, decreased levels of plasma lysophosphatidylcholines (LPCs) were found in both placebo and probiotic groups. However, six other oxidative stress biomarkers including creatine, inosine, hypoxanthine, choline, uric acid, and allantoic acid increased in patients after the two types of therapies. In addition, a positive correlation between changes of LPC (18:0) and Young Mania Rating Scale was found in patients and this association only existed in the probiotic group. Additionally, the mania symptom greatly alleviated in patients who received probiotic supplements as compared with the placebo group. They concluded that the changes in plasma biomarkers of oxidative stress in bipolar disorder patients are trait-like, and can serve as prognostic indexes. The treatment with probiotics may be a promising adjunctive therapeutic strategy for bipolar disorder patients especially in manic episode.

In recent years, the interaction between the bioactive ingredients of traditional Chinese medicine (TCM) and gut microbiota has been a focus of many fields, including oncology and neuropsychiatry, etc. When TCM enters the digestive tract, some ingredients of TCM are metabolized, or bio-transformed by gut microbiota, thereby producing new bioactive molecules, and promote medicine absorption into the circulation. At the same time, the ingredients of TCM impact the composition and abundance of gut microbiota, thereby influencing the remote function of diseased organs/tissues through the systemic action of the gut microbiota (Gong et al., 2020). Herein, Fasina et al. found that gastrodin from *Gastrodia elata* (commonly known as Tian ma in Chinese) enhances cognitive function and neuroprotection of mouse model of Alzheimer's disease via the regulation of gut microbiota. The results showed that gastrodin treatment had a positive correlation with *Firmicutes* and had a negative correlation with *Cyanobacteria, Proteobacteria*, and *Deferribaceters*. Importantly, the lipopolysaccharides and proinflammatory cytokines in the brain increased in Alzheimer's disease mice, but these parameters recovered to normal levels after oral administration of gastrodin. Although detailed mechanisms remain to be clarified, this study added evidence showing gastrodin improves the memory of the Alzheimer's disease mouse model via partly targeting the microbiota-gut-brain axis and mitigating neuron inflammation. Likewise, the combination of TCM may be more effective in treating cognitive impairment as compared with single ingredient from the herb. An optimized combination of TCM named Zi Shen Wan Fang (ZSWF) is composed of *Anemarrhenae Rhizoma, Phellodendri Chinensis Cortex*, and *Cistanches Herba*. Herein, Shi et al. used this prescription for treating diabetes-induced cognitive impairment (DCI) and found that ZSWF restored cognitive function in DCI mice and reduced levels of proinflammatory cytokines such as IL-1β, IL-6, and TNF-α. Moreover, ZSWF protected the integrity of the intestinal barrier by increasing intestinal ZO-1 and occludin protein expression and decreasing urinary lactulose to mannitol ratio, and through reversing the abundance changes of a wide range of intestinal bacteria. In contrast, removing gut microbiota with antibiotics partially eliminated the effects of ZSWF on improving cognitive function and reducing inflammation, confirming the essential role of gut microbiota in the improvement of DCI by ZSWF. These results suggest that ZSWF can be a potential Chinese medicine prescription for DCI treatment.

Despite neuropsychiatric diseases, the importance of the gut-brain axis has been implicated in overall mental health of general population. Although the pandemic of coronavirus disease 2019 (COVID-19) is a fading away recently, the uncertain future, fear of job loss, lockdown and negative news all around have taken a heavy toll on the mental health of individuals from across the world. Stress and anxiety can affect not only the COVID-19 patients but also the ordinary people even more. Here, Dhar briefly summarized the current evidence supporting that gut dysbiosis may be implicated in anxiety and depression both in COVID-19 patients and healthy individuals exposed to various stressors. The author proposed a new perspective indicating that personalized gut microbiome based nutritional strategies, if adopted by people affected by stress and anxiety due to the prevailing environment of COVID-19 and COVID-19 patients themselves, can promisingly improve the mental wellbeing and might act as an alternate mode to assist the mental healthcare infrastructure.

In summary, this collection highlights new findings from original research articles regarding data of both animal experiments and clinical studies, and review articles that help understanding the mechanisms underlying the pathophysiology of gut microbiota played in cognitive deficits, in order to give further insights in the microbiota-oriented treatment for patients with neuropsychiatric disorders and general population affected by stress.

## Author contributions

HC wrote the draft. XC, AB, and RM provided comments for revisions. All authors approved the publication of this editorial.

## References

[B1] Bouvier E. Brouillard F. Molet J. Claverie D. Cabungcal J. H. Cresto N.. (2017). Nrf2-dependent persistent oxidative stress results in stress-induced vulnerability to depression. Mol. Psychiatry. 22, 1701–1713. 10.1038/mp.2016.144 27646262

[B2] Casado A. Castellanos A. López-Fernández M. E. Ruiz R. Imedio E. L. Castillo C.. (2011). Determination of oxidative and occupational stress in palliative care workers. Clin. Chem. Lab. Med. 49, 471–477. 10.1515/CCLM.2011.061 21143019

[B3] Gong X. Li X. Bo A. Shi R. Y. Li Q. Y. Lei L. J.. (2020). The interactions between gut microbiota and bioactive ingredients of traditional Chinese medicines: A review. Pharmacol. Res. 157, 104824. 10.1016/j.phrs.2020.104824 32344049

[B4] Kesika P. Suganthy N. Sivamaruthi B. S. Chaiyasut C. (2021). Role of gut- brain axis, gut microbial composition, and probiotic intervention in Alzheimer's disease. Life Sci. 264, 118627. 10.1016/j.lfs.2020.118627 33169684

[B5] Morkl S. Butler M. I. Holl A. Cryan J. F. Dinan T. G. (2020). Probiotics and the microbiota-gut-brain axis: focus on psychiatry. Curr. Nutr. Rep. 9, 171–182. 10.1007/s13668-020-00313-5 32406013 PMC7398953

[B6] Oliphant K. Allen-Vercoe E. (2019). Macronutrientmetabolism by the human gut microbiome: Major fermentation by-products and their impact on host health. Microbiome 7, 91. 10.1186/s40168-019-0704-8 31196177 PMC6567490

[B7] Perez-Pardo P. Hartog M. Garssen J. Kraneveld A. D. (2017). Microbes tickling your tummy: the importance of the gut-brain axis in Parkinson's disease. Curr. Behav. Neurosci. Rep. 4, 361–368. 10.1007/s40473-017-0129-2 29201595 PMC5694504

[B8] Zeng C. Yang P. Cao T. Gu Y. Li N. Zhang B.. (2021). Gut microbiota: an intermediary between metabolic syndrome and cognitive deficits in schizophrenia. Prog. Neuropsychopharmacol. Biol. Psychiatry 106, 110097. 10.1016/j.pnpbp.2020.110097 32916223

[B9] Zhang T. Yin X. Yang X. Man J. He Q. Wu Q.. (2020). Research trends on the relationship between microbiota and gastric cancer: a bibliometric analysis from 2000 to 2019. J. Cancer 11, 4823–4831. 10.7150/jca.44126 32626529 PMC7330707

